# The unfolded protein response: mechanisms and therapy of neurodegeneration

**DOI:** 10.1093/brain/aww101

**Published:** 2016-05-11

**Authors:** Heather L. Smith, Giovanna R. Mallucci

**Affiliations:** Department of Clinical Neurosciences, University of Cambridge, Cambridge, UK

**Keywords:** neurodegeneration, treatment of dementia, unfolded protein response, neuroprotection, mouse models of neurodegeneration

## Abstract

Activation of the unfolded protein response is emerging as a common theme in protein-misfolding neurodegenerative diseases, with relevant markers observed in patient tissue and mouse models. Genetic and pharmacological manipulation of the pathway in several mouse models has shown that this is not a passive consequence of the neurodegeneration process. Rather, overactivation of the protein kinase RNA-like ER kinase (PERK, encoded by *EIF2AK3*) branch of the unfolded protein response directly contributes to disease pathogenesis through the critical reduction in neuronal protein synthesis rates, essential for learning and memory and for neuronal survival. The pharmacological inhibition of this process in these models is strikingly neuroprotective, resulting in the discovery of the first small molecule preventing neurodegeneration and clinical disease *in vivo*. This now represents a potential generic approach for boosting memory and preventing neurodegeneration across the spectrum of these disorders, albeit with some exceptions, independent of disease-specific proteins. Targeting the unfolded protein response, and particularly PERK-branch mediated translational failure is thus an increasingly compelling strategy for new treatments for dementia and neurodegenerative disease.

## Teaser

Dysregulation of the unfolded protein response is an emerging theme in neurodegenerative disease. Smith and Mallucci present mechanistic insights into this phenomenon from animal models that explain human neuropathological findings. They report that modulation of the pathway can have profound neuroprotective effects, with therapeutic implications for the treatment of dementia.

## Graphical abstract

**Figure:**
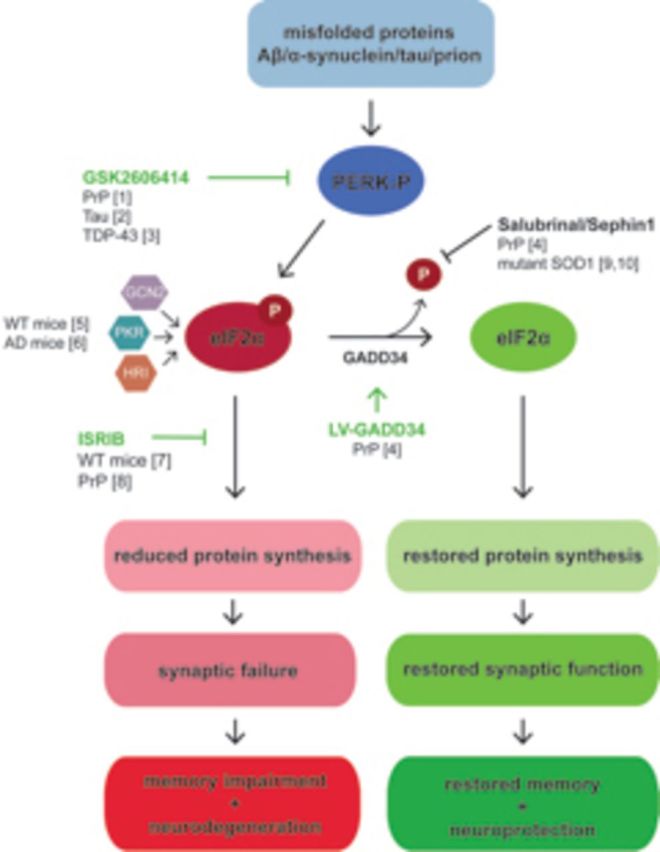
Dysregulation of the unfolded protein response is an emerging theme in neurodegenerative disease. Smith and Mallucci present mechanistic insights into this phenomenon from animal models that explain human neuropathological findings. They report that modulation of the pathway can have profound neuroprotective effects, with therapeutic implications for the treatment of dementia.

## Introduction

Our ageing population will see an ever-increasing prevalence of dementia and neurodegenerative disorders over the coming decades, with imminent rapid escalation in both human suffering and global economic burden. The need to develop effective disease-modifying therapies for these diseases thus represents one of the greatest challenges facing the medical field and society worldwide. Known collectively as the protein misfolding disorders, they include Alzheimer’s and Parkinson’s diseases, frontotemporal dementia and tauopathies, amyotrophic lateral sclerosis (ALS) and the rare prion diseases. Each has a distinct clinical, pathological and biochemical signature, including the accumulation of disease-specific misfolded protein/s in the brain: amyloid-β and hyperphosphorylated tau in Alzheimer’s disease, α-synuclein in Parkinson’s disease, TDP-43 (*TARDBP*), FUS or SOD1 in ALS and prion protein (PrP, encoded by *PRNP*) in prion diseases. How specific misfolded and aggregated protein/s drive neuronal loss in specific diseases remains relatively unclear, and hence disease-modifying therapeutic advances have been elusive to date.

An alternative approach to developing new treatments is to focus on common mechanisms—rather than disease-specific processes—leading to neurodegeneration. The disruption of cellular processes such as autophagy (Menzies *et al.*, 2015) and mitochondrial dysfunction (Burté *et al.*, 2015) contribute to several of these disorders and have been targeted for therapeutic benefit. Most recently, the concept of disrupted protein homeostasis through endoplasmic reticulum (ER) stress and activation of the unfolded protein response (UPR) has emerged as a major common pathogenic theme in neurodegenerative diseases, and a new target for therapy [for reviews see Hetz and Mollereau (2014); Halliday and Mallucci (2015)].

This update will introduce the UPR as a fundamental cellular process for control of protein homeostasis, along with the increasing neuropathological evidence for its dysregulation in human neurodegenerative diseases. It will present recent mechanistic insights from animal models showing that the main driver of neurodegeneration associated with protein misfolding is through activation of a UPR-mediated ‘toxic’ effector pathway, downstream and independent of disease-specific protein. The principal effect of this in many cases is a critical reduction in neuronal protein synthesis rates, essential for learning and memory and for neuronal survival. Further, it will discuss evidence that in several animal models of disease, inhibiting this process therapeutically and restoring neuronal protein synthesis rates represents a generic pathway for boosting memory and the prevention of neurodegeneration that could lead to new treatments across the spectrum of these disorders. It will discuss, models where the opposite is true, but which nonetheless underline the central role played by UPR dysregulation in neurodegenerative disease and the potential for its manipulation in the treatment of these disorders.

## The unfolded protein response

**Figure 1:**
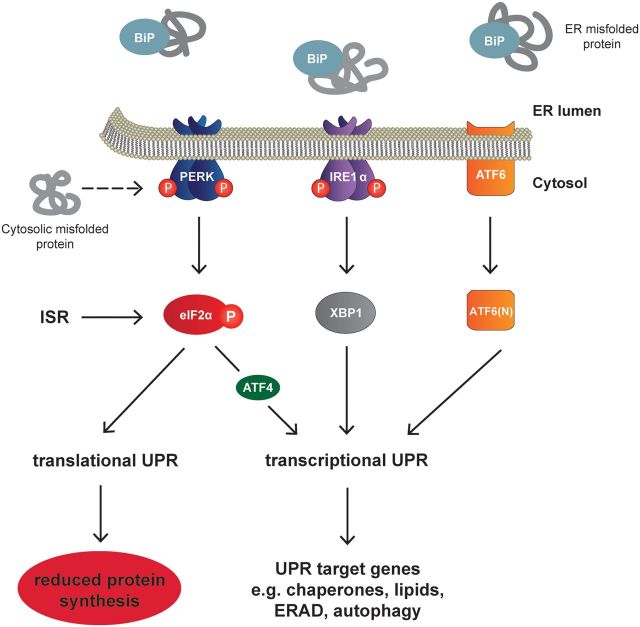
**The unfolded protein response.** Cells have adopted an intrinsic network of protein quality control that promotes efficient protein folding and either the re-folding or degradation of misfolded and aggregated protein. The UPR is one such protective mechanism that acts to restore protein homeostasis (proteostasis) upon the accumulation of misfolded and aggregated protein in the endoplasmic reticulum. The UPR signals through three transmembrane proteins, PERK, IRE1 and ATF6. PERK phosphorylates eIF2α, leading to a rapid attenuation of protein synthesis as well as facilitating the non-canonical translation of *ATF4* mRNA. ATF4 upregulates proteins involved in folding, amino acid metabolism, autophagy and redox balance. eIF2α is also a hub for signalling through the related integrated stress response (ISR), where other kinases phosphorylate eIF2α with resultant translational attenuation and ATF4 upregulation. The IRE1 and ATF6 branches of the UPR activate the transcription factors XBP1 and ATF6 (N), respectively. Target genes encode proteins involved in protein folding, endoplasmic reticulum-associated protein degradation (ERAD) and lipid biosynthesis.

All cells need correctly folded proteins for efficient and accurate functioning. This entails not only the synthesis, but also the carefully regulated folding, trafficking and degradation of individual proteins. When proteins misfold they aggregate and malfunction, which is perceived as a cellular stress. The maintenance of the balance between synthesis and degradation, correct folding and function, is protein homeostasis, or proteostasis. The UPR is a protective mechanism that acts to restore proteostasis in the face of a misfolded protein load (Fig. 1). It does this through a series of transcriptional and translational changes that serve to increase the folding and degradative capacity of the endoplasmic reticulum (Walter and Ron, 2011). The UPR is orchestrated by three transmembrane endoplasmic reticulum proteins; protein kinase RNA (PKR)-like ER kinase (PERK, encoded by *EIF2AK3*), inositol-requiring enzyme 1 (IRE1, encoded by *ERN1*) and activating transcription factor 6 (ATF6). Under resting conditions, the three transmembrane proteins are maintained in an inactive state through their association with binding immunoglobulin protein (BiP, encoded by *HSPA5*), in the endoplasmic reticulum lumen. On the accumulation of unfolded or misfolded protein, BiP dissociates from them and the three sensors are activated. The results of this activation are essentially 2-fold, producing: first, the transient suppression of global protein synthesis rates (translational UPR); and second, the expression of new genes aimed at increasing protein folding and endoplasmic reticulum volume and capacity (via the transcriptional UPR). All three branches have transcriptional effects, but the PERK branch, uniquely, leads to the repression of protein synthesis, essential for learning and memory, synaptic maintenance and neuronal survival. Whilst dysregulation of all three branches has been observed across the spectrum of neurodegenerative disorders, dysregulation of the PERK branch is particularly prominent in human disease, and has marked pathogenic consequences in animal models. The role of PERK-mediated signalling and its modulation in neurodegeneration is therefore the subject of this review. For discussion on the role of IRE1- and ATF6-branch signalling in neurodegenerative disease, see Hetz and Mollereau (2014).

### PERK branch of the unfolded protein response

**Figure 2:**
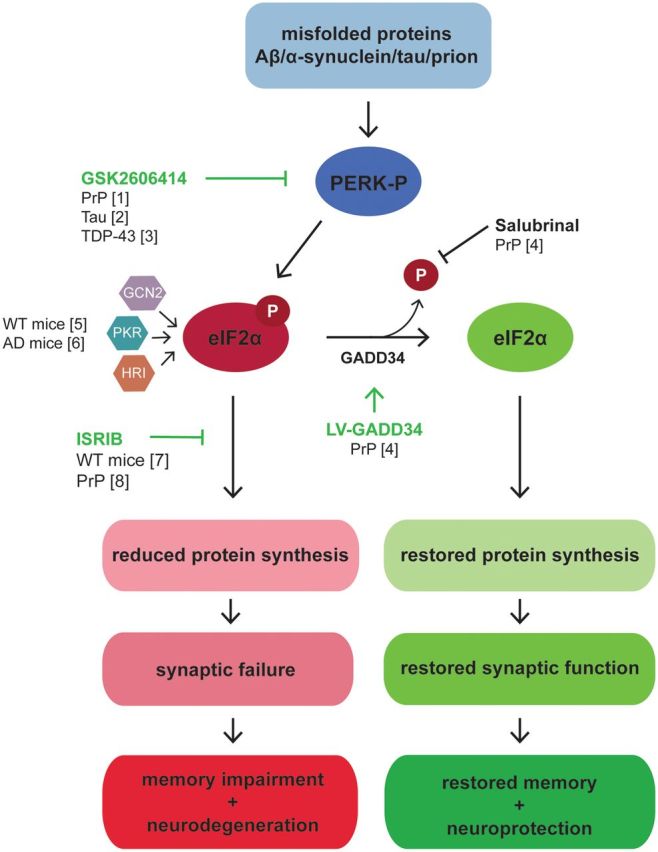
**Genetic and pharmacological inhibition of PERK signalling is neuroprotective in several models of neurodegenerative disease.** The accumulation of misfolded proteins such as PrP and tau results in chronic activation of the PERK branch of the UPR, leading to elevated levels of eIF2α-P. Consequently, there is a sustained reduction in global protein synthesis rates, causing a decrease in the levels of key synaptic proteins, driving synaptic failure, memory impairment and ultimately neuronal loss. In prion-diseased mice, inhibiting the dephosphorylation of eIF2α using salubrinal exacerbates disease, whereas reducing the levels of eIF2α-P through the overexpression of GADD34 is neuroprotective. Pharmacological inhibition of PERK signalling using GSK2606414 reduces PERK-P and eIF2α-P levels in prion-diseased mice, tauopathy mice and in *Drosophila* expressing TDP-43. As a result of this, in the two mouse models, protein synthesis rates are restored, enabling the restoration of synaptic plasticity, memory formation and promoting neuronal survival. Genetic reduction of eIF2alpha-P levels due to PERK or ISR kinase haploinsufficiency restores memory in Alzheimer's disease mouse models. ISRIB restores protein synthesis rates downstream of eIF2α-P and enhances memory formation in wild-type (WT) mice and is neuroprotective in prion-diseased mice. [1] Moreno *et al.*, 2013; [2] Radford *et al.*, 2015; [3] Kim *et al.*, 2014; [4] Moreno *et al.*, 2012; [5] Costa-Mattioli *et al.*, 2007; [6] Ma *et al.*, 2013; [7] Sidrauski *et al.*, 2013; [8] Halliday *et al.*, 2015. Aβ = amyloid-β; AD = Alzheimer’s disease.

PERK is an endoplasmic reticulum transmembrane protein with a cytosolic kinase domain. Following BiP dissociation, PERK dimerizes and autophosphorylates. Activated PERK then phosphorylates the α-subunit of eukaryotic initiation factor 2 (eIF2) in the cytoplasm, preventing the formation of methionine-bearing ternary complex needed to initiate protein synthesis, thereby blocking translation at the level of initiation (Harding *et al.*, 2000) (Figs 1 and 2). By reducing global protein synthesis rates, the PERK branch of the UPR acts to decrease the load of newly synthesized proteins entering the endoplasmic reticulum, thus alleviating any additional burden on the organelle. Although overall global protein synthesis rates are reduced upon the phosphorylation of eIF2α, a number of specific mRNAs are preferentially translated under such conditions (Spriggs *et al.*, 2010). One such mRNA encodes activating transcription factor 4 (ATF4) (Vattem *et al.*, 2004). ATF4 upregulates proteins that function to restore cellular homeostasis, such as endoplasmic reticulum chaperones and proteins involved in amino acid metabolism and redox control (Bravo *et al.*, 2013). Critically, ATF4 also upregulates GADD34 (*PPP1R15A*), the inducible regulatory subunit of protein phosphatase 1 (PP1) that promotes the dephosphorylation of eIF2α, providing a negative feedback mechanism that allows the restoration of protein synthesis rates. If endoplasmic reticulum stress is unresolved, prolonged expression of ATF4 upregulates the pro-apoptotic factor C/EBP homologous protein (CHOP), which initiates the apoptotic signalling cascade and cell death.

## PERK branch activation in neurodegenerative disease

### PERK branch unfolded protein response markers in neuropathology of human disorders

Over the past decade, histological evidence of UPR activation, particularly the detection of phosphorylated PERK (PERK-P) and eIF2α-P, has increasingly been reported in post-mortem brain tissue from patients with Alzheimer’s disease, ALS, Parkinson’s disease, and also in the tauopathies progressive supranuclear palsy and frontotemporal dementia (Hoozemans *et al.*, 2007, 2009; Atkin *et al.*, 2008; Stutzbach *et al.*, 2013). The upregulation of PERK branch UPR markers in these disorders is both temporally and spatially associated with the accumulation of misfolded and aggregated protein (Roussel *et al.*, 2013; Stutzbach *et al.*, 2013). In Alzheimer’s disease, UPR activation is thought to occur early in the aggregation process, with BiP and PERK-P levels increasing in association with phosphorylated tau, prior to neurofibrillary tangle formation (Hoozemans *et al.*, 2009; Nijholt *et al.*, 2012). A recent genome-wide association study searching for common variants influencing the risk of progressive supranuclear palsy identified a single-nucleotide polymorphism in intron 2 of the PERK gene *EIF2AK3* (Höglinger *et al.*, 2011). Patient-derived lymphoblastoid cell lines showed a stronger UPR stress response than controls (Liu *et al.*, 2012), suggesting that the variant may increase the risk of this tauopathy via over-activation of the UPR. However, the pathological significance of this activation in human disease remains largely unknown: whether it reflects a protective mechanism or actively contributes to disease pathogenesis is unclear from neuropathological observations alone.

### Deleterious effects of PERK signalling in prion-diseased mice

Animal models of neurodegeneration also exhibit signs of endoplasmic reticulum stress. Raised levels of PERK-P and eIF2α-P are observed in prion-diseased mice (Unterberger *et al.*, 2006; Moreno *et al.*, 2012; Halliday *et al.*, 2015); in Tg4510 mice, which overexpress the frontotemporal dementia-associated human tau mutation P301L (Abisambra *et al.*, 2013; Radford *et al.*, 2015); in 5xFAD mice that express five Alzheimer’s disease-linked mutations (Devi and Ohno, 2014); and in mutant SOD1-expressing mice (Saxena *et al.*, 2009). Thus, both human post-mortem tissue and mouse models of neurodegenerative diseases show a correlation between UPR (particularly PERK branch) markers and neuropathology.

Insight into the mechanistic relevance of these observations comes from recent work in prion-diseased and frontotemporal dementia mice. Moreno and colleagues (2012) provided the first demonstration that chronic PERK signalling is detrimental to neuronal survival and drives neurodegeneration in prion-diseased mice (Fig. 2). They found that the accumulation of misfolded PrP during disease, leads to chronically elevated levels of PERK-P and eIF2α-P, resulting in the sustained reduction in global protein synthesis rates in the brain. Such persistent translational repression occurs in the context of already compromised neuronal function (due to already established synapse loss) and the resulting catastrophic decline in levels of key synaptic proteins leads to further synaptic failure and ultimately neuronal loss and demise of the animal. Crucially, restoring protein synthesis rates by genetic manipulation of the pathway to reduce levels of eIF2α-P prevented neurodegeneration and clinical disease in prion-infected mice (Moreno *et al.*, 2012) (Fig. 2). Thus, both over-expressing GADD34 to reduce the levels of eIF2α-P directly through its dephosphorylation, or knocking down levels of PrP to remove the source of UPR activation and hence of elevated eIF2α-P, promoted translational recovery, with restoration of global protein synthesis rates. As a result, synaptic protein levels, synaptic transmission and synapse number in the prion-diseased mice were protected and indeed restored to levels seen in uninfected control mice. Neuronal protection was observed throughout the hippocampus of prion-infected animals, with no neuronal loss and markedly reduced spongiform degeneration. In each case, the protection was seen despite ongoing prion replication and accumulation. Conversely, treating prion-diseased mice with the small molecule salubrinal, which effectively blocks the inactivation of eIF2α-P, accelerated clinical disease and neuronal loss and shortened survival. The data thus not only demonstrate that eIF2α-P-mediated translational repression is a critical mediator of neurodegeneration in prion disease, but also establish an important proof of principle, that preventing the phosphorylation of eIF2α represents a valid therapeutic approach in this model.

## Restoring protein synthesis rates for therapy of neurodegeneration

### Pharmacological inhibition of PERK signalling prevents clinical disease and neurodegeneration

**Figure 3:**
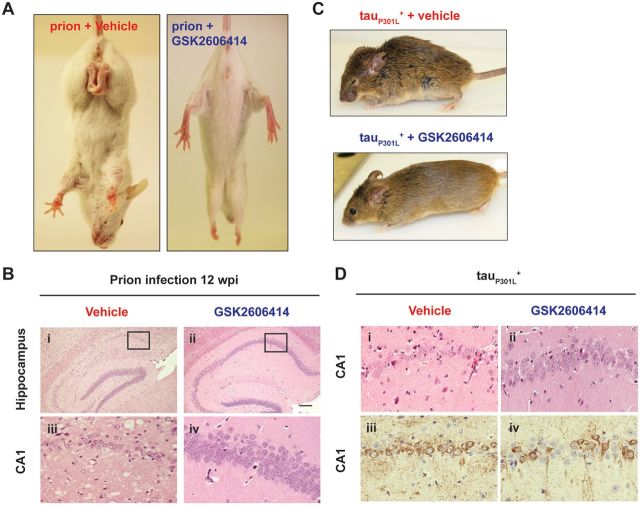
**PERK inhibition by GSK2606414 prevents clinical disease and neuronal loss in prion-diseased and frontotemporal dementia mice.** (**A**) Prion-diseased mice were treated with vehicle or GSK2606414 from 7 weeks post-infection (wpi). At 12 weeks post-infection, GSK2606414-treated mice lacked the clinical signs of prion disease, with normal posture and movement of hind legs. (**B**) At 12 weeks post-infection, GSK2606414-treated prion-diseased mice showed marked neuroprotection in the hippocampus. (**C**) GSK2606414-treated frontotemporal dementia mice show normal grooming, posture and movement compared to vehicle-treated mice. (**D**) GSK2606414 treatment resulted in marked neuroprotection in the frontotemporal dementia mice, with the preservation of hippocampal neurons (i–ii). Immunostaining showed reduced levels of phosphorylated tau in the GSK2606414-treated mice (iii–iv).

These data led to the prediction that pharmacological inhibition of PERK signalling to reduce eIF2α-P levels would be neuroprotective in these mice. Prion-diseased mice treated orally with the compound GSK2606414, a highly selective inhibitor of PERK, showed reduced levels of PERK-P and eIF2α-P, independent of upstream prion replication. The compound restored protein synthesis rates and, at 12 weeks post-infection, when all vehicle-treated mice had succumbed to clinical prion disease, all GSK2606414-treated mice were free of the clinical signs of prion disease, with neuroprotection observed throughout the brain (Fig. 3A and B) (Moreno *et al.*, 2013). This PERK inhibitor was thus the first small molecule to prevent neurodegeneration *in vivo*. Critically, it targeted not the specific pathogenic protein (PrP in this case), but a generic downstream process of protein misfolding.

PERK/eIF2α-P-mediated translational repression has also been identified as a key mediator of neuronal loss in a mouse model of frontotemporal dementia (Abisambra *et al.*, 2013; Radford *et al.*, 2015). Tg4510 mice, which overexpress the human tau mutation P301L, show elevated levels of eIF2α-P and ATF4 at 6 months of age (Radford *et al.*, 2015). At this time point, protein synthesis rates, which are normal at 5 months, decline to ∼40% of those seen in non-transgenic littermate controls. As with prion-diseased mice, the onset of eIF2α-P-mediated translational repression is closely associated with the onset of neurodegeneration, with tau_P301L_^+^ mice exhibiting memory impairment and hippocampal neuronal loss from 6 months of age. Here too, treatment with the PERK inhibitor GSK2606414 from 6 months significantly reduced the levels of PERK-P, eIF2α-P and ATF4, restoring protein synthesis rates and protecting against further neuronal loss (Fig. 3) (Radford *et al.*, 2015). As a result, GSK2606414 treatment reduced brain atrophy and abrogated the appearance of clinical symptoms in tau_P301L_^+^ mice. PERK inhibition also reduced the levels of phosphorylated tau, with markedly lower levels of pathological tau staining in the brains of GSK2606414-treated tau_P301L_^+^ mice (Fig. 3C and D). PERK inhibition therefore acts via two mechanisms to protect against tau-mediated neurodegeneration, by restoring vital protein synthesis rates and by decreasing the phosphorylation, and likely the associated toxicity, of tau.

A similar approach may also be relevant in sporadic ALS. In a fly model of the disease, Bonini and colleagues observed a progressive increase in the levels of eIF2α-P in *Drosophila* expressing TDP-43 (Kim *et al.*, 2014). When fed GSK2606414, TDP-43-expressing flies showed markedly lower levels of eIF2α-P, with a dramatic mitigation of TDP-43-induced climbing dysfunction and motor weakness.

### Fine tuning the restoration of protein synthesis rates: balancing risk and benefit

Inhibiting eIF2α-P-mediated translational repression thus represents a promising therapeutic approach in several neurodegenerative diseases including prion, tauopathies and sporadic ALS. However, before this is pursued for drug development, it is important to consider the extent to which protein synthesis rates should be restored. In prion-diseased and frontotemporal dementia-tauopathy mice, GSK2606414 treatment restored protein synthesis rates to ∼100% (Moreno *et al.*, 2013; Radford *et al.*, 2015). Unfortunately, while being profoundly neuroprotective, the complete inhibition of PERK signalling is toxic to the pancreas, where a degree of eIF2α-P-mediated translational repression is essential to cope with the high secretory load of the tissue (Moreno *et al.*, 2013; Halliday *et al.*, 2015). Similar phenomena are also seen in PERK-deficient mice (Harding *et al.*, 2001) and in the rare human disorder Wolcott-Rallison syndrome, which presents with neonatal diabetes due to loss-of-function mutations in the PERK gene *EIF2AK3* (Julier and Nicolino, 2010). Another solution is therefore needed to achieve a balance between avoiding complete inhibition of a vital pancreatic endoplasmic reticulum stress response and restoring protein synthesis rates to compromised neurons. The pancreas, with its huge secretory protein synthetic burden presents a very different cellular environment to that found in ageing, diseased neurons. Already compromised by dysfunctioning mitochondria, autophagy and deficient repair processes and with critically reduced synapse number, these are exquisitely sensitive to further reductions in synaptic proteins. In these neurons, inhibiting PERK overactivation and restoring protein synthesis rates allows recovery of synapse function and number and enables learning and memory. In pancreas, however, it prevents the necessary lull in protein synthesis rates for chaperones and related processes to deal with a misfolded protein load. For therapy, a compromise is needed.

The small molecule inhibitor, ISRIB, which acts to restore protein synthesis downstream of eIF2α-P, a point of convergence of the UPR and the related integrated stress response (ISR) (Fig. 1), improves memory in wild-type mice without reported toxic side effects *in vivo* (Sidrauski *et al.*, 2013) (see below). Crucially, in prion-diseased mice, ISRIB treatment confers neuroprotection without pancreatic toxicity, a likely consequence of ISRIB restoring protein synthesis rates to ∼70% of levels seen in uninfected control mice (Halliday *et al.*, 2015), rather than 100% as for GSK2606414. Importantly, this ‘partial’ restoration of protein synthesis rates is sufficient to protect neurons and increase the life span of ISRIB-treated prion-diseased mice, while allowing sufficient capacity for the pancreas to handle endoplasmic reticulum stress. Fine-tuning the extent of UPR inhibition and subsequent translational de-repression is therefore able to uncouple neuroprotective effects from pancreatic toxicity and is the target for therapy. While ISRIB itself is not a candidate for modulating eIF2α-P, due to its high insolubility, it again establishes an important proof-of-principle and highlights the need for partial rather than complete de-repression of protein synthesis for the development of safe therapeutics.

### Added benefits: boosting protein synthesis rates boosts memory and learning

The aberrant phosphorylation of eIF2α has also been implicated in memory loss, a key early clinical feature in many neurodegenerative diseases. Long-term synaptic plasticity and memory formation relies on *de novo* protein synthesis; the sustained phosphorylation of eIF2α therefore impinges on this process. Further, ATF4 is a repressor of cAMP response-element binding protein (CREB)-mediated gene expression, which is essential for synaptic plasticity and learning (Chen *et al.*, 2003). In mice, reducing the phosphorylation of eIF2α via the heterozygous expression of an eIF2α phospho-null mutant lowers the threshold for long-term potentiation, enhancing synaptic plasticity, spatial learning and memory (Costa-Mattioli *et al.*, 2007). Similarly, Ma *et al.* (2013) showed that the genetic deletion of PERK and GCN2 (an ISR eIF2α kinase, see Figs 1 and 2) improved synaptic plasticity and spatial memory in a mouse model of Alzheimer’s disease. Pharmacologically, the small molecule ISRIB, which inhibits signalling downstream of eIF2α-P, enhances spatial and fear-associated learning in wild-type mice (Sidrauski *et al.*, 2013). Conversely, blocking the dephosphorylation of eIF2α has detrimental effects of cognitive function, impairing both late long-term potentiation and long-term memory in mice (Costa-Mattioli *et al.*, 2007) and accelerating memory loss in prion-diseased mice (Moreno *et al.*, 2012). Mice expressing human APOE4, the main risk factor for sporadic Alzheimer’s disease, also show increased levels of eIF2α-P, further supporting the link between translational repression and cognitive impairment (Segev *et al.*, 2013). Thus, preventing eIF2α-P-mediated translational repression therapeutically will likely also protect against early synaptic failure and memory loss in Alzheimer’s disease and related disorders, as well as later neuronal demise.

### Exceptions and caveats

We have discussed how dysregulated PERK signalling is detrimental to neuronal survival in the context of PrP, mutant tau and TDP-43 aggregation, and to learning and memory in both health and disease models, as well as the association of PERK-P and eIF2α-P with progression of Alzheimer’s disease. There are examples of neurological disorders, however, where signalling via the PERK branch of the UPR is not deleterious (Table 1). In mutant SOD1 mouse models of ALS, activation of the PERK branch appears to mediate a pro-survival response (Saxena *et al.*, 2009; Das *et al.*, 2015), similarly in a A53T alpha-synuclein mutation model of Parkinson’s disease (Colla *et al.*, 2012). The same is seen in mice modelling the rare demyelinating peripheral neuropathy Charcot–Marie–Tooth disease type 1b (CMT1b) (D’Antonio *et al.*, 2013; Das *et al.*, 2015). In these cases, prolonging rather than inhibiting eIF2α-P signalling is neuroprotective. In SOD1 G93A mice, sustaining the phosphorylation of eIF2α using the drug salubrinal delays disease progression, counteracting the loss of muscle force and extending life-span (Saxena *et al.*, 2009). Salubrinal is also protective in A53T alpha-synuclein mice (Colla *et al.*, 2012). Treatment with Sephin1, a small molecule inhibitor of the regulatory subunit of the eIF2α-P phosphatase PP1, similarly results in reduced mutant SOD1 aggregation and motor neuron degeneration in the same mice (Das *et al.*, 2015). Consistent with these findings, the hemizygous deletion of PERK in another familial ALS mouse model (SOD1 G85R) is detrimental, accelerating disease onset and shortening life-span, except in later disease, when it is protective (Wang *et al.*, 2011)—possibly due to the greater influence of factors affecting ageing neurons later in disease, discussed above.

It is interesting to postulate on why inhibiting the PERK branch signalling can be protective in some neurodegenerative disorders yet exacerbate disease in others. Such conflicting observations may be explained by the nature of stress produced by the misfolded proteins in the different models. In some rare genetic forms of ALS and Parkinson’s disease, mutant SOD1 and mutant α-synuclein are suggested to lead to a ‘pure’ form of endoplasmic reticulum stress, due to their accumulation within the endoplasmic reticulum. Similarly, in CMT1b (a disorder primarily of peripheral nerve Schwann cells), the P0 mutant protein is retained in Schwann cell endoplasmic reticulum with resultant endoplasmic reticulum stress. In these cases, prolonging eIF2α-P signalling both reduces synthesis of the mutant protein and increases the time for refolding through chaperone expression, representing a beneficial protective response. Another hypothesis is that mutant SOD1 proteins cause endoplasmic reticulum stress through retrograde signalling to the endoplasmic reticulum, where reducing protein synthesis through prolonged eIF2α-P signalling allows this to be alleviated by restoring proteostasis within the endoplasmic reticulum.

However, in the majority of neurodegenerative disorders, including Alzheimer’s disease, idiopathic Parkinson’s disease and sporadic ALS, various tauopathies and the prion diseases, the accumulating misfolded proteins are mainly cytoplasmic and are largely not produced in the endoplasmic reticulum. Therefore, the dysregulation of proteostasis is not ‘true’ endoplasmic reticulum stress in these diseases. In fact, exactly how PERK is overactivated is unclear—likely involving a non-canonical as yet unexplained mechanism of activation. Whatever the exact mechanism, the resultant chronic PERK-eIF2α-P signalling is detrimental to the cell not through conventional endoplasmic reticulum stress, but through sustained deprivation of essential protein synthesis in metabolically compromised, aged neurons. Here, translational shutdown is a major lethal driving force behind neurodegeneration; hence inhibiting eIF2α-P signalling is protective.

**Table 1:** Effects of genetic and pharmacological interventions on eIF2α-P levels on disease progression in mouse models of protein misfolding neurodegenerative diseases

Disease	Model	Misfolded protein	Manipulation	Effect on eIF2α-P levels	Outcome	Reference
Alzheimer’s disease	APP/PSEN1 mice	Amyloid-β	Deletion of PERK/GCN2	Decreased	Rescued memory deficits	Ma *et al.*, 2013
	5XFAD mice	Amyloid-β	PERK haploinsufficiency	Decreased	Rescued memory deficits and neuroprotective	Devi and Ohno, 2014
Amyotrophic lateral sclerosis	SOD1 G93A mice	Mutant SOD1	Salubrinal	Increased	Neuroprotective	Saxena *et al.*, 2009
			Sephin1	Increased	Neuroprotective	Das *et al.*, 2015
	SOD1 G85R mice	Mutant SOD1	PERK haploinsufficiency	Decreased	Accelerated disease	Wang *et al.*, 2011
	Drosophila	TDP-43	GSK2606414	Decreased	Neuroprotective	Kim *et al.*, 2014
Parkinson’s disease	A53T mice	α-Synuclein	Salubrinal	No change reported	Delayed symptom onset	Colla *et al.*, 2012
Frontotemporal dementia	Tau P301L mice	Tau	GSK2606414	Decreased	Neuroprotective	Radford *et al.*, 2015
Prion disease	Prion-infected mice	PrP	Overexpression of GADD34	Decreased	Neuroprotective	Moreno *et al.*, 2012
			Knockdown of PrP	Decreased	Neuroprotective	Moreno *et al.*, 2012
			GSK2606414	Decreased	Neuroprotective	Moreno *et al.*, 2013
			ISRIB	Acts downstream of eIF2α-P	Neuroprotective	Halliday *et al.*, 2015
			Salubrinal	Increased	Accelerated disease	Moreno *et al.*, 2012
No disease	Wild-type mice	-	ISRIB	Acts downstream of eIF2α-P	Improves memory	Sidrauski *et al.*, 2013
			Salubrinal derivative	Increased	Impairs memory	Costa-Mattioli *et al.*, 2007

## Concluding remarks

In conclusion, whilst PERK branch signalling may have opposite effects in different protein misfolding disorders, the evidence from these many different models highlights the central pathogenic role of this pathway across the spectrum of these disorders and its tractability for manipulation—in either direction—for the prevention of neurodegeneration in a number of different disorders. In particular, in the majority of these models, including tauopathy—hence with broad relevance, particularly to Alzheimer’s disease—restoration of protein synthesis rates due to dysregulated PERK signalling is a powerful neuroprotective strategy. Given the current global crisis in new cases of dementia, the pursuit of careful targeting of this process has real potential for translation into new treatments for neurodegenerative disease.

## Funding

G.R.M. is funded by the Medical Research Council and a European Research Council Consolidator award holder.

## Abbreviations

ALS: amyotrophic lateral sclerosis
eIF2: eukaryotic initiation factor
PERK: protein kinase RNA-like ER kinase
UPR: unfolded protein response
